# Capability of insulator study by photoemission electron microscopy at SPring-8

**DOI:** 10.1107/S0909049513012508

**Published:** 2013-05-30

**Authors:** Takuo Ohkochi, Masato Kotsugi, Keisuke Yamada, Kenji Kawano, Koji Horiba, Fumio Kitajima, Masaki Oura, Susumu Shiraki, Taro Hitosugi, Masaharu Oshima, Teruo Ono, Toyohiko Kinoshita, Takayuki Muro, Yoshio Watanabe

**Affiliations:** a Japan Synchrotron Radiation Research Institute/SPring-8, 1-1-1 Kouto, Sayo, Hyogo 679-5198, Japan; b Institute for Chemical Research, Kyoto University, Gokasho, Uji, Kyoto 611-0011, Japan; c Materials Reseach and Developement Division, Taiyo Yuden Co. Ltd, 5607-2 Nakamuroda, Takasaki, Gumma 370-3347, Japan; d Department of Applied Chemistry, The University of Tokyo, 7-3-1 Hongo, Bunkyo-ku, Tokyo 113-8656, Japan; e Synchrotron Radiation Research Organization, The University of Tokyo, 7-3-1 Hongo, Bunkyo-ku, Tokyo 113-8656, Japan; f Department of Earth and Planetary Sciences, Kyushu University, 6-10-1 Hakozaki, Higashi-ku, Fukuoka 812-8581, Japan; g RIKEN SPring-8 Center, 1-1-1 Koto, Sayo, Hyogo 679-5148, Japan; h WPI-Advanced Institute for Materials Research, Tohoku University, 2-1-1 Katahira, Aoba-ku, Sendai, Miyagi 980-8577, Japan; i JST-ERATO, Keio University, 3-2-1 Sakado, Takatsu-ku, Kawasaki, Kanagawa 213-0012, Japan

**Keywords:** PEEM, insulator, ferrite, evaporator, photon-induced surface conductivity

## Abstract

The observation method of photoemission electron microscopy (PEEM) on insulating samples has been established in an extremely simple way. Surface conductivity is induced locally on an insulating surface by continuous radiation of soft X-rays, and Au films close to the area of interest allow the accumulated charges on the insulated area to be released to ground level. Magnetic domain observations of a NiZn ferrite, local X-ray absorption spectroscopy of sapphire, high-resolution imaging of a poorly conducting Li_0.9_CoO_2_ film surface, and Au pattern evaporation on a fine rock particle are demonstrated. Using this technique, all users’ experiments on poorly conducting samples have been performed successfully at the PEEM experimental station of SPring-8.

## Abstract 2

A photoemission electron microscopy measurement system on insulating samples was established at the BL17SU beamline of SPring-8 by utilizing an Au pattern evaporation technique combined with photon-induced surface modification.

## 1. Introduction   

Photoemission electron microscopy (PEEM) in the soft X-ray region is a powerful tool for nanoscale science (Guo *et al.*, 2007 ▶). In recent years, demands for nanospectroscopic studies on insulating materials such as oxides, semiconductors, resins and extra-terrestrial matter have been rapidly increasing from beamline users at the PEEM experimental station of BL17SU in SPring-8. Although, unlike scanning probe microscopy, PEEM does not have an *ultimate* spatial resolution, it possesses an *appropriate* resolution and many useful functions such as chemical mapping, element-specific magnetic domain observation, X-ray absorption spectroscopy and X-ray photoemission spectroscopy on the nanoscale. In addition, unlike transmission X-ray microscopy, PEEM does not require thin-film-shaped samples. Thus, PEEM experiments are desired by many synchrotron radiation users from a variety of research scopes, in spite of the fact that poor electric conductivity of samples, which causes surface charging, is formidable for photoelectron measurements. Up to now, we have made a novel discovery of rare-metal-free hard magnets, L1_0_-type FeNi, by means of magnetic domain observations of meteoric irons (Kotsugi *et al.*, 2010 ▶) and are now planning to extend our PEEM studies to insulating meteorites. In particular, micrometre-sized particles recovered from the asteroid Itokawa by the Hayabusa mission are targets of great interest for exploring the origins of space (for recent reports on investigations of Itokawa samples, see Tsuchiyama *et al.*, 2011 ▶; Nakamura *et al.*, 2011 ▶; Yurimoto *et al.*, 2011 ▶; *etc.*). Hence, establishing ways to investigate insulating materials with PEEM should be quite important from the viewpoint of developing new scopes in applied physics.

Some strategies for solving the charging problem in photoelectron measurements have already been reported. In X-ray photoemission electron spectroscopy, electron flooding guns have been conventionally used to stabilize the surface electrical potential (Kelly, 2010 ▶). However, with this method it is not easy to establish a fully compensated surface potential and the photoelectrons emitted by the injected electrons sometimes form an inhomogeneous background. As another method, it is sometimes effective to deposit *in situ* a very thin metallic film on the whole sample, monitoring the image by PEEM in real time. However, the covering film often becomes too thick to investigate the target elements in detail, while the conductivity of the whole surface is not sufficient for establishing a fully compensated surface charge.

In this paper we report on a technique to observe insulators by PEEM established at the synchrotron radiation facility SPring-8 by means of an Au pattern deposition method combined with a local surface modification effect by continuous irradiation of synchrotron radiation. This method is quite simple and effective compared with those reported so far. We introduce some examples of PEEM studies using this technique, such as area-selective X-ray absorption spectroscopy of sapphire and magnetic domain observations of NiZn ferrite. The method is also useful for stabilizing the electric potential of the surface of samples such as Li_0.9_CoO_2_ thin films that are not insulating but poorly conducting, and for marking the position of small rock particles (50–100 µm) buried in insulating plates.

## 2. Experimental   

The experiments were performed at the PEEM experimental station of beamline BL17SU (a-branch) of SPring-8 (Ohashi *et al.*, 2007 ▶; Senba *et al.*, 2007 ▶). In this beamline, circularly or linearly polarized soft X-rays (200–2000 eV) generated from a multi-polarization-mode undulator are available (Shirasawa *et al.*, 2004 ▶; Oura *et al.*, 2007 ▶) and the X-rays are focused to a diameter of ∼30 µm by a condensing mirror just before the PEEM experimental station. We employed a spectroscopic photoemission/low-energy-electron microscope (SPELEEM; Elmitec Co. Ltd), achieving a spatial resolution of ∼23 nm even in PEEM experiments in the soft X-ray region (Guo *et al.*, 2007 ▶). All experiments were performed under a pressure of 3 × 10^−9^ to 5 × 10^−9^ torr and at room temperature.

**Figure 1:**
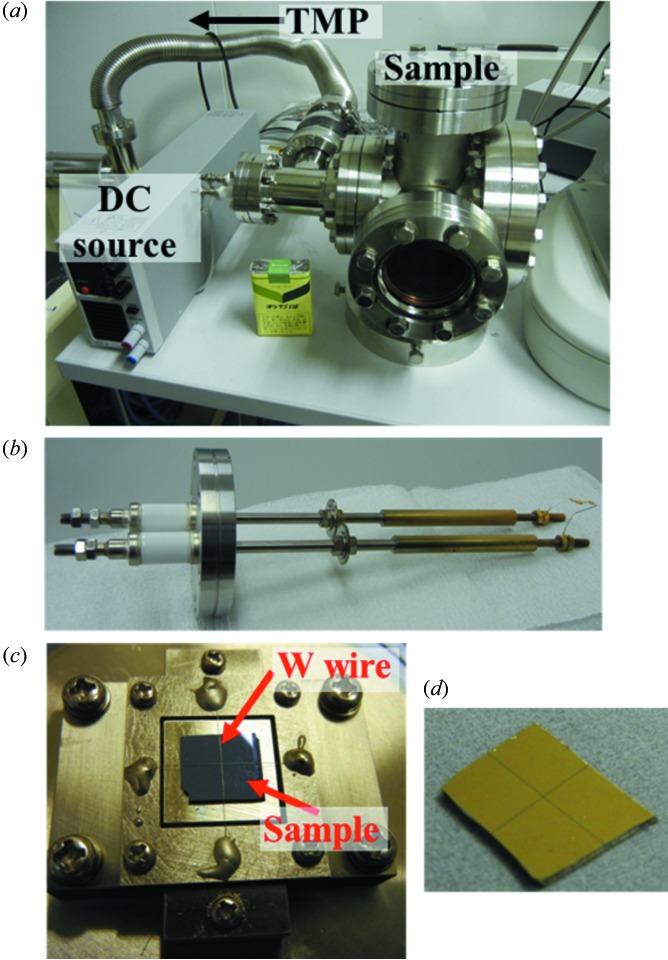
(Colour online) Photographs of (*a*) the Au pattern evaporator, (*b*) the evaporation source, (*c*) the Si substrate fixed by tungsten wire and (*d*) the Si substrate with Au patterned film.

In order to effectively release the positive charges created by photoemission, we deposited *ex situ* thick (typically ∼100 nm) Au films just around the observation area. Fig. 1(*a*) ▶ shows a compact Au evaporator composed of simple instruments: an evaporator [a DC source and a tungsten filament; Fig. 1(*b*) ▶], a turbomolecular pump (TMP) and a sample holder. To screen the area of interest in the sample, we used tungsten wires stretched on a Cu frame (Fig. 1*c* ▶). The width of the exposing area can be selected by using wires of different thickness. This method is more useful than using commercial metal masks to deposit Au on the whole surface except for very limited areas. We show in Fig. 1(*d*) ▶ an example of a Si substrate with Au patterned film grown by this method. This type of evaporating system can be introduced in any experimental facility at low cost; in this method, metals with high steam pressure such as Au can be thickly deposited by resistance heating, since the metal is not deposited in the area of interest, whereas, in the conventional method of growing a very thin film on the whole surface, wetting metal such as Pd or Ru must be evaporated using high-power sources. Au films were evaporated quickly under low vacuum, ∼1 × 10^−2^ to 10^−3^ Pa. A series of sample treatments, *i.e.* sample mounting, pumping, evaporating and vending, can be performed within ∼1 h.

## 3. Results and discussions   

**Figure 2:**
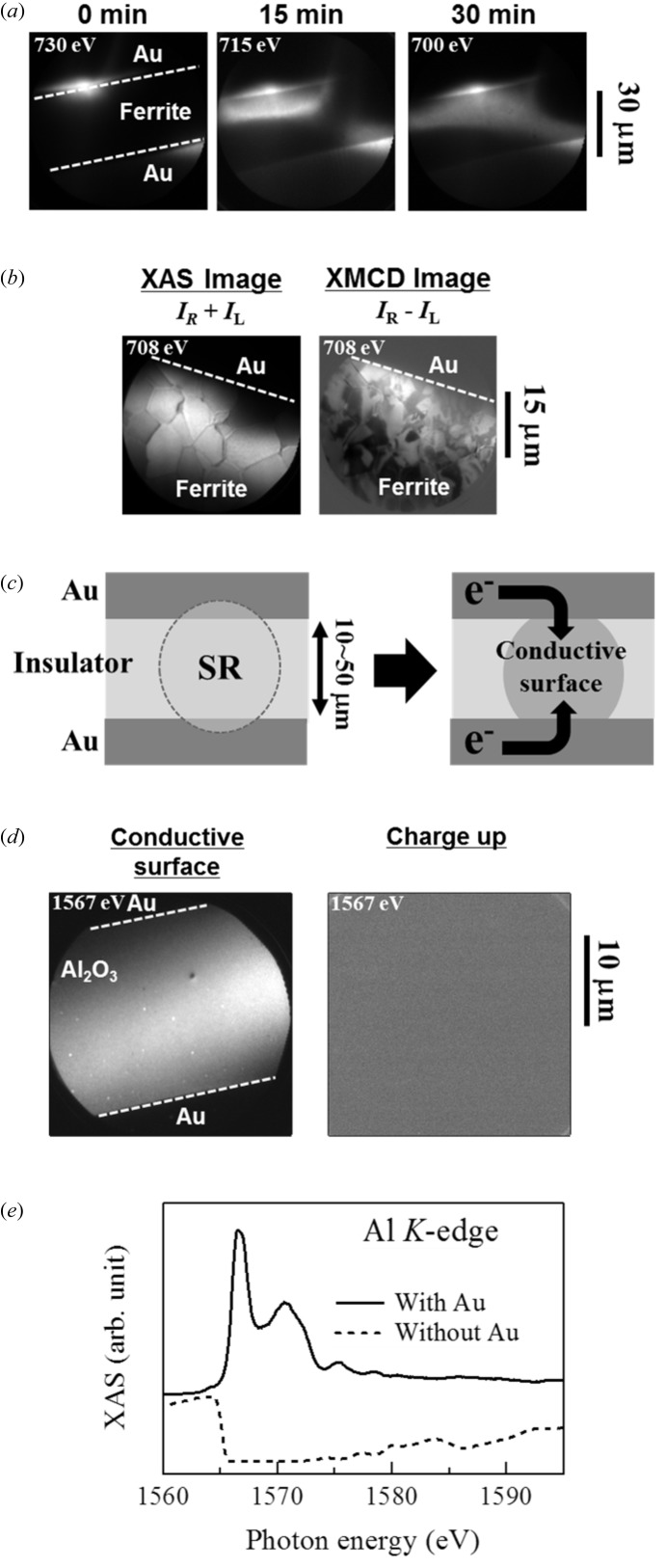
(*a*) PEEM images of NiZn ferrite surrounded by Au pads during the course of radiation of soft X-rays around the Fe *L*_3_-edge (730–700 eV) for 0–30 min. (*b*) XAS and XMCD images of NiZn ferrite at the absorption peak of the Fe *L*_3_-edge (708 eV) after irradiation of synchrotron radiation for ∼30 min. (*c*) Schematics of an insulating sample surrounded by thick Au pads and its process of obtaining electric conductivity by synchrotron radiation irradiation. (*d*) PEEM images at the Al *K*-edge (1567 eV) and (*e*) XAS spectra around the Al *K*-edge of a sapphire substrate after (solid line) and before (dotted line) irradiation of synchrotron radiation for 1 h.

Fig. 2(*a*) ▶ shows PEEM images of a sintered polycrystalline NiZn ferrite (∼1 kΩ cm) covered by thick Au except for a thin area of width ∼30 µm. This sample has been thermally etched in order to remove the residual stress induced by the surface polishing and no magnetic field is applied before the PEEM experiments (for more details, see Kawano *et al.*, 2009 ▶). The images were recorded during an X-ray absorption spectroscopy (XAS) scan around the Fe *L*_3_-absorption edge (730–700 eV) with ∼7 × 10^12^ photons mm^−2^ s^−1^ irradiated on a bare ferrite surface. Just after starting the radiation (‘0 min’ in the figure) the electric charges appear to accumulate at the surface and no image is obtained from the ferrite. However, by maintaining the synchrotron radiation illumination, the images from the ferrite gradually become visible from the boundary with the Au films. After 30 min of synchrotron radiation illumination, good electric conductivity is ensured in all irradiated areas and image contrast is clearly seen. Fig. 2(*b*) ▶ shows XAS-PEEM [image (right circular polarization) + image (left circular polarization), *I*_R_ + *I*_L_] and XMCD-PEEM (*I*_R_ − *I*_L_) images in the same area as in Fig. 2(*a*) ▶ but with an expanded field of view, taken at the absorption peak of the Fe *L*_3_-edge (708 eV). One can see from the XAS image (chemical contrast) that this NiZn ferrite is composed of crystal grains with sizes of about 5–10 µm. As is clear from the XMCD image, the ferrite forms random magnetic domains and most of their domain sizes are smaller than those of crystal grains. Moreover, the magnetic domain boundaries are formed independent of the position of the crystal boundaries. This fact is clarified for the first time by using PEEM microscopy; in the case of imaging by a magneto-optical Kerr microscope, the image contrast mostly came from chemical compositions or different crystalline orientations between grains, so that the magnetic domain structure could not be recognized. A detailed XMCD-PEEM study on NiZn ferrites with various grain sizes will be reported elsewhere (Kawano *et al.*, 2013 ▶).

We show schematically in Fig. 2(*c*) ▶ a possible process for the insulator surface to become electrically conductive. Continuous illumination by high-flux X-rays enhances surface conductivity due to photo-induced surface modification, and then electric charges are supplied from the surrounding Au pads. The present method serves to detect weak signals from a target element, since *no* capping metal is covering the observation region. Even though local surface conductivity may be occurring in every synchrotron radiation experiment, we should again emphasize that the thick Au films play the roll of an electric pathway between the reconstructed insulator surface and the ground level. It should also be noted that intermixing of materials of interest with capping metals (Ohno *et al.*, 1993 ▶; Kröger *et al.*, 2005 ▶) can be avoided using this method. The effects of photoradiation such as damage, surface reconstruction or photo-induced surface conductivities have been discussed for a long time. The origin of enhanced conductivity in our experiment does not seem to be the plasma charging effect which sometimes occurs in experiments with photons in the vacuum ultraviolet region (for details, see Cismaru *et al.*, 2000 ▶), but is likely to be an irreversible process such as surface damage or the accumulation of a very thin contamination layer (Gilbert *et al.*, 2000 ▶). The influence of surface damage on samples is discussed later.

By applying the same method, even genuine insulators such as Al_2_O_3_ (∼10^14^ Ω cm at room temperature) can be investigated by PEEM. The left-hand panel of Fig. 2(*d*) ▶ shows a PEEM image at the Al *K*-edge (1567 eV) of a sapphire substrate covered by Au with a narrow window (width 20 µm) after the irradiation of soft X-rays of ∼2 × 10^12^ photons mm^−2^ s^−1^ for 1 h. Detailed topological structure derived from fine contamination particles is distinctly seen. In Fig. 2(*e*) ▶ we show a local XAS spectrum of sapphire around the Al *K*-edge in the observation area (solid line). This spectrum shows sharp absorption peaks and has quite a similar shape to that of Al_2_O_3_ obtained using a crystal spectrometer (Kinoshita *et al.*, 1998 ▶), indicating that the PEEM investigation can be performed without charge accumulation and modification of the surface state does not seem to be caused by radiation damage to Al_2_O_3_ itself. For comparison, a similar image [right-hand panel of Fig. 2(*d*) ▶] and spectrum [dotted line in Fig. 2(*e*) ▶] of the same sample before exposing to sufficient synchrotron radiation are also shown. No image contrast can be obtained under this condition. At energies higher than ∼1566 eV in the XAS spectrum shown in Fig. 2(*e*) ▶, far from observing absorption peaks the intensity become rather lower, because increased X-ray absorption enhances charge accumulation.

**Figure 3:**
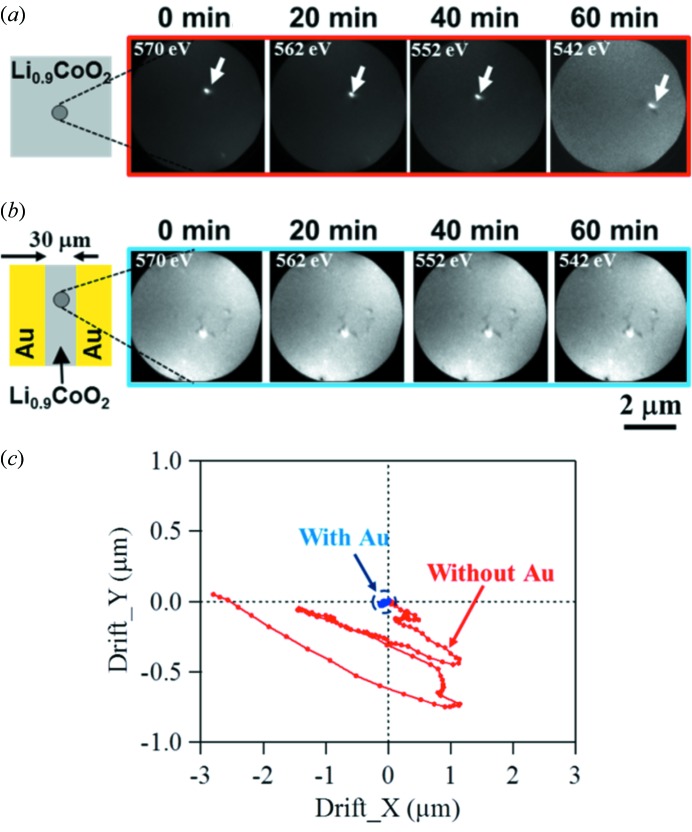
(Colour online) (*a*) PEEM images of Li_0.9_CoO_2_ film during the course of an XAS scan around the O *K*-edge (570–530 eV) for ∼60 min. (*b*) Similar images to those in (*a*) but of Li_0.9_CoO_2_ film covered by Au pads. (*c*) Plots of displacements of PEEM images on Li_0.9_CoO_2_ samples with and without Au pads during the course of PEEM observations around the O *K*-edge for ∼80 min.

The Au patterned pads not only work as electric paths but are also useful for stabilizing the surface electric field of poorly conducting samples. Fig. 3(*a*) ▶ shows PEEM images of Li_0.9_CoO_2_ thin film grown on an Al_2_O_3_(0001) substrate without Au pads at representative photon energies around the O *K*-edge (570–530 eV). Li_0.9_CoO_2_ is a typical battery electrode material with an electric resistivity of several hundred Ω cm at the surface. Generally, photoemission spectroscopic or microscopic measurements on such samples are possible to some extent, and, in fact, noticeable image contrast can be obtained as seen in this figure. Nevertheless, the position of a defect (indicated by white arrows) is unstable because the surface potential is not fully compensated and the trajectory of photoelectrons varies, connected to the amount of accumulated charge. Fig. 3(*b*) ▶ shows similar images to Fig. 3(*a*) ▶ but the Li_0.9_CoO_2_ is covered by Au pads except for a thin area of width 30 µm. Topological contrast can be seen in detail compared with those in Fig. 3(*a*) ▶ and practically no image drift is noticed. We summarize in Fig. 3(*c*) ▶ the displacements of the PEEM images of Li_0.9_CoO_2_ samples with and without Au pads during the course of the XAS scan for ∼80 min. Without Au, the image moves ∼3 µm at the most. However, by adding Au pads in the surrounding area, the displacement is reduced to ∼±10 nm. This value can be attributed to slight mechanical drift of the sample manipulator. Note that, in cases of samples whose electric conductivities are similar to that of this sample, clear images can be obtained without inducing local conductivity by exposing X-rays for some time, since the charging effect is mostly wiped out by stabilizing the macroscopic electric potential of the whole surface.

**Figure 4:**
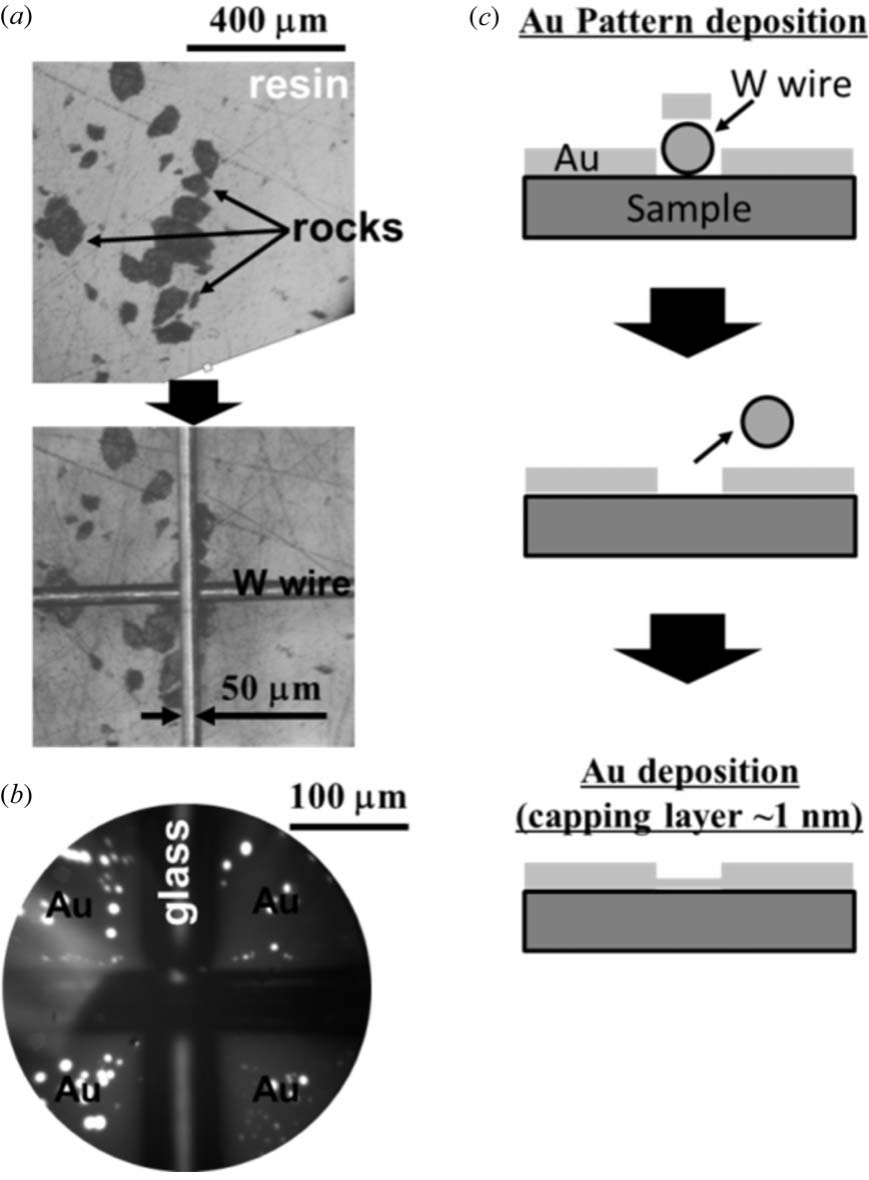
(*a*) Stereoscopic microscope images of 50–100 µm-sized rock particles buried on resin before and after masking with tungsten wires. (*b*) Cross-lined pattern (Au on glass substrate) seen in the field of view of PEEM with a UV lamp (diameter ≃ 300 µm). (*c*) Schematic images of Au pattern deposition on the PEEM sample followed by evaporation of a ∼1 nm-thick protection layer.

We also show a good example of this technique working as a target marker. Fig. 4(*a*) ▶ shows stereoscopic microscope images of 50–100 µm-sized standard rock particles buried in a resin before Au deposition. By monitoring with a wide-field microscope, tungsten wires can be appropriately positioned in the sample plate with an accuracy of ∼10 µm even by hand, as shown in the lower panel of Fig. 4(*a*) ▶. The cross-line-shaped bare insulator is clearly recognized by the UV-PEEM (PEEM with mercury lamp) observation because the working function of insulating materials is quite different from that of Au (Fig. 4*b* ▶). Therefore, this pattern helps us to find out the target material even in a narrow field of view of PEEM (diameter 300–500 µm at most). We have actually applied this technique to a *single* particle (typically ∼50 µm diameter) recovered from the asteroid Itokawa. The results of micro-XAS experiments on this sample will be reported in another paper.

We mentioned above that the local electronic conductivity on the insulator surface is enhanced by photon-induced surface modification. In many cases this may be attributed to contamination derived from residual gas molecules (C atoms, oxides and so on) which turned into radicals by strong X-rays and accumulated on the irradiated area. While it is necessary to consider possible radiation damage, at most ∼30–60 min of synchrotron radiation is needed for the insulating surface to gain good conductivity. Samples are irradiated for 1–2 h in a fixed area even in many conventional PEEM experiments with *conductive* samples, yet one does not need to discuss the damaging effect in many cases. In other words, insulators can be investigated with a similar exposure time to the case of conducting samples. However, in some cases of soft materials such as organic materials, samples are destroyed by synchrotron radiation immediately and our present method is no longer applicable as it is. For such materials we additionally deposit ∼1 nm of Au protection layer after making thick Au pads, as schematically shown in Fig. 4(*c*) ▶. Although Au covers the whole surface by this treatment, the surrounding thick Au pads are still important to neutralize the surface charge. This method is similar to one reported earlier (Stasio *et al.*, 2003 ▶); however, with our system, thin Au film can be evaporated with good accuracy, simplicity and reproducibility, by putting a calculated amount of Au source on the filament and evaporating it completely [the relation between the volume of the Au source (*V*_source_) and the thickness of the Au film (*t*_Au_) is expressed as *t*_Au_ = (*d*_bulk_ *V*_source_)/(*d*_film_4π*r* ^2^), where *d*_bulk_ and *d*_film_ denote the volume densities of bulk Au and Au film, respectively, and *r* is the distance between the deposition source and sample substrate].

While the main target of PEEM studies in our earlier works had been metallic samples such as magnetic materials, demands by beamline users for PEEM investigations of insulating samples continue to increase year after year. Since the last half of 2010 when the present method was introduced, experimental subjects on insulators have reached ∼43% (13 proposals out of 30), including studies on ferrites, meteorites, biological samples, polymers and so on. Our technique has aided in charging problems in all these subjects and will play an important role in elucidating the microscopic electronic states of a variety of materials of interest. For example, space-resolved XAS analysis of dielectric materials with their electronic/magnetic states modified by applied electric field is now in process. Other studies are also planned such as analysis of biological samples and magnetic domain imaging of rocks containing fine magnetic minerals which maintain ancient magnetization directions. In order to clarify the mechanism of as yet unidentified surface modifications, detailed analysis of thin conductive layers created by photon radiation (*i.e.* local XAS analysis at the C and O *K*-edges) is desired. Systematic experiments under various chamber pressures and quantitative experiments using samples whose surface resistivity is definitely specified are also important subjects.

## 4. Summary   

We have established a way to investigate insulating samples with PEEM by making the insulator surface exposed in a thin-wired shape using an Au pattern evaporator. The local electric conductivity on the insulator surface can be obtained by radiating strong soft X-rays for 30–60 min, due to the photon-induced surface modification. This technique enables us to obtain photoemission signals from the target element quite effectively compared with the conventional method of thin-metal deposition. The instruments used for this method can be introduced to any experimental stations with ease and at low cost. We have demonstrated the magnetic domain observation of NiZn ferrite, and the high-resolution imaging and micro-XAS of Al_2_O_3_ and Li_0.9_CoO_2_. By precisely adjusting the position of the masking wires, even rare and microscopic particles such as extra-terrestrial matters can be investigated. This technique has been utilized by a variety of beamline users at BL17SU in SPring-8.
